# Automatic Detection of Magnetic Disturbances in Magnetic Inertial Measurement Unit Sensors Based on Recurrent Neural Networks

**DOI:** 10.3390/s23249683

**Published:** 2023-12-07

**Authors:** Elkyn Alexander Belalcazar-Bolaños, Diego Torricelli, José L. Pons

**Affiliations:** 1 Neural Rehabilitation Group, Cajal Institute, Spanish National Research Council (CSIC), 28002 Madrid, Spain; 2 Department of Automation and Systems Engineering, Carlos III University, 28911 Madrid, Spain; 3 Legs and Walking AbilityLab, Shirley Ryan AbilityLab, Chicago, IL 60611, USA; 4 Department of Physical Medicine and Rehabilitation, Feinberg School of Medicine, Northwestern University, Evanston, IL 60208, USA; 5 Department of Biomedical Engineering, McCormick School of Engineering and Applied Science, Northwestern University, Evanston, IL 60208, USA; 6 Department of Mechanical Engineering, McCormick School of Engineering and Applied Science, Northwestern University, Evanston, IL 60208, USA

**Keywords:** inertial measurement, magnetic disturbance, magnetometer, deep neural

## Abstract

This paper proposes a new methodology for the automatic detection of magnetic disturbances from magnetic inertial measurement unit (MIMU) sensors based on deep learning. The proposed approach considers magnetometer data as input to a long short-term memory (LSTM) neural network and obtains a labeled time series output with the posterior probabilities of magnetic disturbance. We trained our algorithm on a data set that reproduces a wide range of magnetic perturbations and MIMU motions in a repeatable and reproducible way. The model was trained and tested using 15 folds, which considered independence in sensor, disturbance direction, and signal type. On average, the network can adequately detect the disturbances in 98% of the cases, which represents a significant improvement over current threshold-based detection algorithms.

## 1. Introduction

In the past three decades, human motion analysis has witnessed great scientific advances related to microelectromechanical system (MEMS) technologies. In this respect, the inertial measurement unit (IMU) stands out, mainly because of its high accuracy and low cost. Walking speed assessment [1], gait evaluation [2], pedestrian dead reckoning [3], and activity classification [4] are some of the primary applications.

An IMU fuses physical data obtained from sensors of different natures and then reports information such as dynamic acceleration, angular rate, or, in most cases, orientation. A basic configuration considers six degrees of freedom (DOF) using accelerometer and gyroscope sensors. Although those sensors may be sufficient to estimate orientation, some errors arise depending on the physical phenomena from each sensor technology, e.g., offset error, drift, misalignment, noise, or environmental sensitivity.

Most estimating methods solve these problems by adopting three DOF or more through magnetometers, known as magnetic IMU (MIMU). The magnetometer and accelerometer compensate the drift that occurs during the integration of the gyroscope data, thereby giving precise 3D orientation [5,6,7]. The orientation estimation process exploits the properties of both the Earth’s gravitation and magnetic fields, which are widely known physical phenomena. The attitude and tilt angles can be estimated by computing the angle with respect to the vertical direction, assuming that the gravity is much greater than the external acceleration. However, considering gravity alone is insufficient to determine the angle around the vertical axis, i.e., the heading angle. To address this issue, the physical properties of the Earth’s magnetic field are utilized, particularly its horizontal component.

Even though the Earth’s magnetic field is a valuable aid to having an absolute orientation reference, the presence of ferromagnetic materials distorts the reference and, therefore, the orientation.

Many researchers have focused on characterizing the magnetic disturbances, mostly in indoor applications. The relationship between the distance and the magnetic source was shown to have the first impact on inertial magnetic orientation sensors [8]. In another approach [9], the magnetic disturbance’s spatial spread was mapped, thus finding that the homogeneous magnetic field has a critical dependency with the floor distance. In [5,10], the authors classified the distortion effects in hard and soft iron. The former is related to permanently magnetized objects, and the latter is related to objects magnetized by an external field. Both distortions can be corrected by a sensor calibration process only when the magnetic environment does not change. However, magnetic fields with variations in space or time remain challenging to work with.

The literature considers several solutions to this challenge. The simplest solution establishes a decision criterion when the magnetometer signal is reliable, defining a threshold in its magnitude [11,12]. Limiting the magnetometer contribution to the heading variable is another common method [13]. A widely used method even isolates up to two components [6]. In addition, a novel solution suggests a model-based estimation of the disturbance. For example, ref. [14] considers that the magnetic field direction is estimated simultaneously with the sensor orientation. Another approach [6] assumes that the magnetic field is constant for a given period when using a magnetometer reading taken at the start of a period as a point of reference. The authors updated the error state calculated in their Kalman filter using the error pertaining to this reference at each time step. The accuracy of these methods relies on detecting the disturbance by thresholding or modeling.

In summary, one of the biggest challenges regarding dealing with magnetic disturbance in the orientation estimation process is detecting the magnetic disturbances accurately. We have noticed that the main detection methods consider thresholding or magnetic field modeling. However, these methods proved to be insufficiently robust. Therefore, we propose a new methodology for the automatic detection of magnetic disturbances based on deep learning, specifically using recurrent neural networks (RNNs). The paper is organized as follows: Section 2 provides a brief description of the background of wearable sensor systems and the main approaches to deal with magnetic disturbances. Section 3 introduces the main concepts regarding deep learning methods. Section 4 describes the experimental methodology based on the reproducible generation of controlled magnetic fields and the design, training, and testing of the proposed network. Section 5 reports the performance of the proposed RNNs, and Section 6 discusses the obtained results and the limitations of this work. Section 7 summarizes the conclusions.

## 2. Background

A wearable sensor system (WSS) is a device that enables the monitoring of human activities through an array of sensors installed on a person’s body. Typically, these devices are used to register such activities over long periods of time [15,16]. The most common applications of WSSs are the monitoring of biomechanical [17,18,19] and movement [20,21,22,23,24] parameters. In the context of physiological monitoring, WSSs have shown to be useful to detect pathologies [25] and to better plan therapy treatments for patients with neurological, cardiovascular, or pulmonary diseases [26].

A large body of the literature has been devoted to investigating human movement under different perspectives, such as movement mechanisms [27,28,29,30], rehabilitation [31,32,33], robotic aids [34,35,36,37], or exercising with the help of a virtual trainer [38,39,40]. The development of MEMS technology, and in particular the MIMU sensors, led to small, light, and affordable solutions to analyze human movement.

In the following paragraphs, the principles of the MIMUs are described, considering the principal electronic components and their main issues.

### 2.1. Basic Principles and Common Issues in MIMU Technology

**Figure 1:**
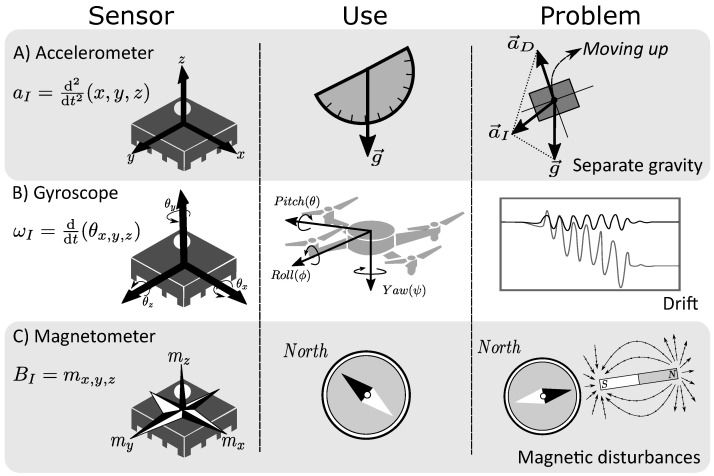
Comparison of MIMU sensors. In the first column, the physical principles and their mathematical representations are compared; in the next column, the main use of each sensor is presented: the accelerometer allows us to estimate the gravity vector; the gyroscope is used to estimate the rotation angles; and the magnetometer gives the reference of the Earth’s magnetic field. In the last column, we show the common issues: (**A**) separate gravity in dynamic conditions; (**B**) drift due to intrinsic gyroscope properties; and (**C**) magnetic disturbances.

MIMUs are often used to estimate the position and/or orientation of body parts. To do so, the properties of each sensor are exploited through sensory fusion strategies. Nonetheless, there are limitations to each technology that are important to understand. As shown in Figure 1, the sensors that measure the magnetic field operate on the Lorentz force principle to move mechanical parts. The magnetic field induces a current in the conductive loops, which moves under the influence of the Lorentz force and proportionally to the strength of the magnetic field.

The magnetometers, like the gyroscopes and accelerometers, suffer from their own implementation challenges. A crucial factor influencing the long-term durability of magnetometers is their temperature sensitivity. Common approaches have computed corrections and a temperature-stable environment to increase precision [41]. Typically, manufacturers include an embedded temperature sensor, which greatly improves the performance of temperature compensation algorithms.

Magnetometer sensors are also sensible to surrounding electronics and circuitry, which can distort the measurements of the electromagnetic field (EMF). This problem can be caused even by the internal circuitry of the MIMU or its battery. Some of these disturbances, i.e., the stationary ones, can be mitigated with calibration protocols [42,43]. External elements in the environment can also cause interference in the magnetic readings, such as ferromagnetic materials (passive disturbance) or electronic devices (active disturbance). Isolating the effects of such external factors is a challenging and unsolved problem.

Sensory integration methods [5,6,44,45] integrate the angular velocity over time to determine the orientation, thereby causing errors to grow rapidly due to the drift of the gyroscope. For this reason, this approach is mostly suitable for short-term estimation.

Combining the information provided by the accelerometers and the magnetometers is a means to mitigate the drifts of the gyroscope. In typical implementations, the accelerometer measures the inclination of the body, and the magnetometer measures the magnetic field, which serves as a compass that provides the orientation of the body. The assumption that these two variables are always measurable only holds under static conditions—that is, when the MIMU is not moving. If the MIMU is moving, on the contrary, the accelerometer will measure both gravitational and dynamic accelerations.

### 2.2. Dealing with Magnetic Disturbances

Prior knowledge about the behavior of the Earth’s magnetic field, such as its strength and the dip angle, makes it possible to reject disturbed magnetic measurements. A straightforward and computationally efficient solution is to define precise threshold values. However, threshold adjusting is typically problematic, and for magnetic features near the threshold value, the algorithm’s behavior can become chaotic. Some examples are provided in the following.

In [11], measurements from an accelerometer and a magnetic sensor are used to adjust quaternion predictions made using a gyroscope. Before updating the correction, they checked the magnetic output sensor. Both the magnetic field norm and dip angle were compared to thresholds. When at least one of the differences exceeded the respective threshold, they discarded the magnetic data. If the magnetic measurement was approved, the noise covariance matrix was used to set the confidence of the readings.

In [12], the authors proposed using a linear Kalman filter (KF). Using the quaternion from the quaternion estimator (QUEST) algorithm, they updated the quaternion predicted by the gyroscope in the presence of magnetic disturbances, thus discarding the information from the magnetometer. A similar case was considered in [11], where the authors compared both magnetic and accelerometer sensors with thresholds. They proposed to estimate the prediction values of the magnetic field and gravity. Then, they used them as an input in the QUEST algorithm if the measurement was discarded instead of the sensed magnetic field.

Another approach to reject magnetic disturbances is model-based. It considers that the magnetic reading is systematically disturbed by undesired elements, thereby adding inaccurate and unexpected components in the Earth’s magnetic field. The objective is to estimate and model magnetic disturbances at each filtering algorithm iteration step in order to compensate for the unprocessed model. Compared to threshold-based methods, the computation power increases because the state vectors augment with extra components. Two main methods have been proposed in the literature.

The first method was presented in [14], which assumes first-order Gauss–Markov (GM) dynamics. They used three additional state components of the indirect Kalman filter (IKF) to estimate magnetic disturbance magnitude. The magnetic sensor output was compensated prior to being employed in updating the anticipated state vector. However, they compared a threshold method with the magnetic field predicted by the extended Kalman filter (EKF) to assess the magnetic data reliability.

In [46], a variable-state dimension EKF was proposed. Then, they considered the first-order GM dynamics under low or null magnetic disturbance conditions. In high-disturbance conditions, a second-order GM model was built by adding three more states to the state vector until a low magnetic disturbance condition was detected. The authors detected disturbances from the difference between the expected and measured fields.

In [47], a linear KF was first used to isolate the Earth’s magnetic field and magnetic disturbances. Then, both conditions were directly included within the state vector and predicted through the gyroscope data and a first-order GM model, respectively. Finally, the sum of the two magnetic vectors defined the magnetic sensor through the linear and time-invariant measurement model, and the final orientation estimation was produced. This approach’s contribution lies in postponing the linear framework to a second step, where a KF took place.

Both threshold- and model-based methods rely on the accurate detection of the time instant when the magnetic disturbance occurs. This information is used to reject or compensate the disturbance in the orientation estimation. However, both methods have shown issues in this respect. In threshold-based methods, the accuracy decreases when the threshold is close to the Earth’s magnetic field or when the threshold is estimated during dynamic changes to the magnetic field. In model-based methods, the detection algorithm is trained to learn specific conditions where it works correctly. Otherwise, the model fails in new spaces.

In order to solve these problems, we propose to use deep neural networks, which have demonstrated excellent performance on complex classification tasks in many areas. The following section provides a brief description of the main principles and approaches in deep learning, which are useful for understanding our proposed methodology.

## 3. Deep Learning Methods

In recent years, deep learning has gained significant success and public interest due to its ability to extract meaningful patterns from high-dimensional unstructured data, such as speech recognition [48,49], acoustic modeling [50,51], trajectory prediction [52], sentence embedding [53], and correlation analysis [54]. Recently, some works also focused on detecting magnetic anomalies [55,56].

Deep learning techniques are based on neural networks, which are mathematical representations of how the brain uses several levels of abstraction to interpret input data and arrive at a conclusion. Neural networks learn complex computation, even though they are related to traditional statistical models.

Standard feedforward neural networks are designed for independent data points only. Recurrent neural networks (RNNs) are particularly useful for processing time series or sequence-based data, where one data point depends on the preceding data point(s). In order to produce the next output in the sequence, RNNs use the idea of memory to retain the states or data of earlier inputs. However, some issues arise when modeling extended temporal sequences with long-term dependencies.

Some approaches addressed these issues by integrating signals with varying time constants [57] or by introducing leaky units [58]. Nonetheless, another way has stood out over them, the long short-term memory (LSTM) units, which have proven to be more effective in solving the training issues with RNNs.

### Long Short-Term Memory (LSTM)

LSTM is a type of RNN that is capable of learning long-term dependencies [59]. In contrast to standard neural networks, LSTM allows loops in its network, because these loops are able to remember past events and then optimize the input data actions. This method handles long-range dependencies, thus highlighting many sequential data tasks. However, a standalone LSTM is suitable for a sequence-based task, where the input is a sequence, but the output is not. For cases with sequential-type inputs and outputs, it is possible to use a sequence-to-sequence method.

Sequence-to-sequence LSTM is a specific use case that involves two RNNs together: an encoder and a decoder. The fundamental concept underlying this architecture is to transfer an arbitrary-length input sequence to an output sequence that may differ in length from the input sequence.

## 4. Materials and Methods

In this section, we describe the deep learning architecture that we proposed for the automatic detection of magnetic disturbances. We also explain the experimental protocol that we used to train and test this model. Furthermore, we present the details of the experimental validation process and the performance analysis indicators.

### 4.1. Sequence-to-Sequence LSTM Model

**Figure 2:**
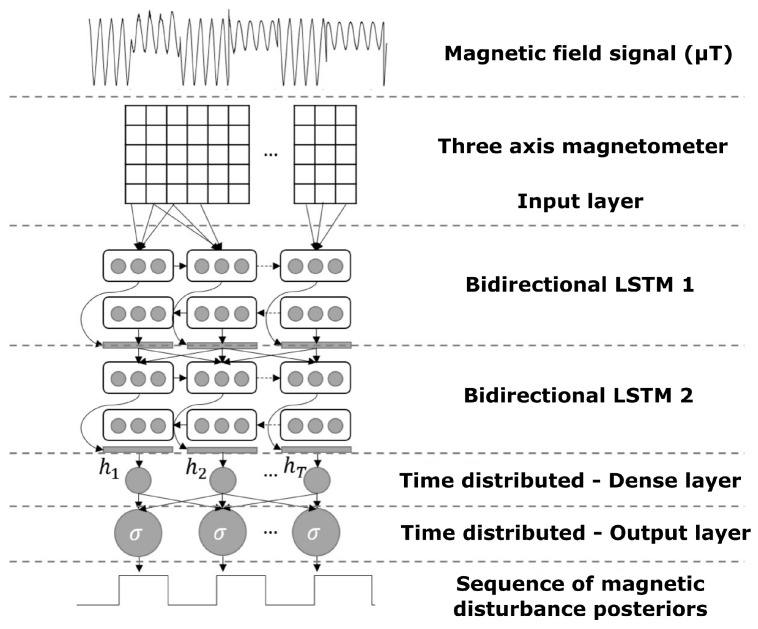
Neural network architecture to estimate the magnetic disturbance posteriors from magnetometer signal.

We proposed a model to predict the magnetic disturbance probabilities, which was applied to magnetometer data recorded at 100 Hz. The model receives as input the three-axis magnetometer signal, without windowing. Figure 2 shows the step-by-step procedure of the architecture used to train the neural network.

Each sample is a feature sequence for the neural network’s input layer. Then, we chose two bidirectional LSTM layers. These layers process feature sequences from the input while simultaneously modeling information from the past (backward) and future (forward) states of the sequence.

The output sequence of the second bidirectional LSTM layer passes through a hidden dense layer of time division, thereby maintaining a one-to-one relationship between the lengths of the input and the output sequences, i.e., we applied a fully connected dense layer at each time step, producing an output sequence with exactly the same input length.

To produce the final sequence of probabilities, we connected the time-distributed hidden layer to the time-distributed output layer using a softmax activation function. This function is suitable for converting a vector of *N* real numbers into a probability distribution with the same number *N* of possible outcomes.

We trained the networks using a cost function. A crossentropy cost function compares the predicted probabilities with the true probabilities of the target, thus avoiding the unbalance of the classes. See Equation (1):

(1) $$\iota =\sum_{i=1}^{C}w_{i}p_{i}log\left(\hat{p_{i}}\right)$$

The percentage of samples from the training set that belong to each class defines the weight factors $w_{i}$ for each class i=1…C. We optimized the networks using the Adam method. Finally, to improve the generalization of the proposed networks, we considered dropout layers, which are able to correct overfitting, and batch normalization layers, thereby keeping the mean output close to zero and the output standard deviation close to one.

### 4.2. Experimental Protocol for Data Generation

**Figure 3:**
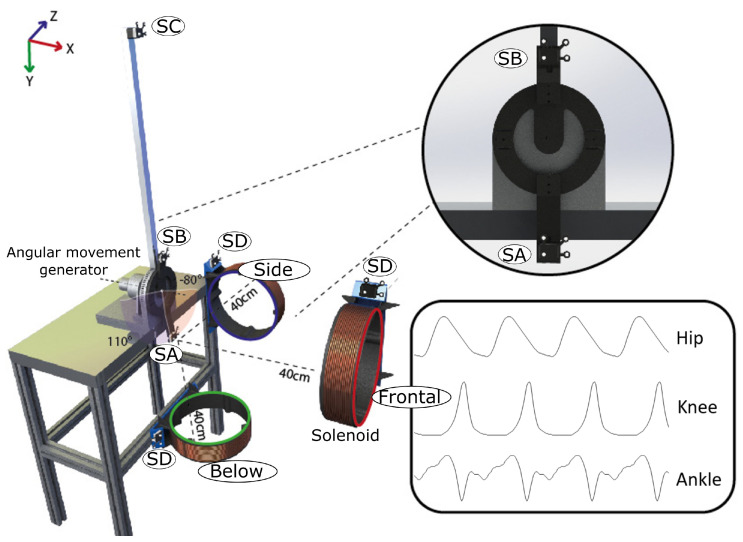
Experimental setup. We used a 1-DOF device that can produce varying angular positions, four MIMU sensors (SA and SB are fixed to the joint axes, SD is also connected to the Solenoid to label the magnetic disturbances states, and SC is located away from the experiment to monitor possible external anomalies), and one solenoid to produce a magnetic disturbance. The right box shows some examples of the signals we used: hip, knee, and ankle in the sagittal plane.

We conducted a series of repeatable and reproducible experiments with four 9-DOF MIMU sensors from Technaid company. We exposed the sensors to various magnetic perturbations in both static and dynamic scenarios to collect data for the classification model. The experiment aims to detect the ability of the proposed sequence-to-sequence LSTM method to detect the presence of magnetic disturbances. In order to produce the controlled magnetic field, we used a custom electromagnet based on a solenoid coil. To generate movement in the MIMU sensors, we used an angular movement generator (electrical motor) attached to a bar, on which IMU sensors were placed. Figure 3 shows the complete setup.

**Figure 4:**
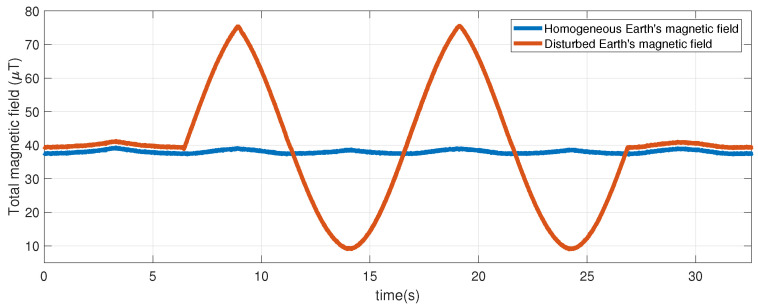
Change in the amplitude of the magnetic disturbance. The blue plot shows the magnetic field free of magnetic disturbance. The orange graph shows how the amplitude of the magnetic field disturbance varies as a function of the distance to the solenoid.

Each solenoid was able to generate an artificial magnetic field with values between 40 and 200 microteslas (μT). Although each solenoid was only manipulated by turning the power supply on and off, the magnetic field disturbance varied depending on the relative distance and orientation between the MIMU sensor and the axis of the solenoid, which varied according to the angular position of the bar on which the sensor was attached. Figure 4 shows the amplitude changes in the magnetic disturbance due to the change in distance between the sensor and the solenoid.

Knowing that the electrical motors used to apply motion to the MIMU sensors generate a magnetic field when a current is applied, we ensured a distance where the magnetic flux did not affect the measurement of the inertial sensors. To check this, we used a total of four sensors: two sensors were used to collect the artificial magnetic field Sensor A closest to the axes joint and Sensor B farthest from the joint axes). Another sensor (Sensor C) was placed far enough from the experiment, thereby ensuring that there were no other external sources of magnetic disturbances. One additional sensor (Sensor D) was attached to the active solenoid to check the ON–OFF condition of the induced magnetic field.

Taking advantage of the mechanical design of the joint, the protocol considers typical signals of human movement, particularly for the lower body: the sinusoidal signal; flexion–extension for the hip, knee, and ankle; and a step-by-step signal changing every three degrees between −80° to 110°. The patterns were reproduced 20 times each, with a time length between 10 and 200 s. Over these signals, we automatically turned on the solenoid, thereby disturbing the signals in a controlled way and considering long and short time disturbances. Finally, to affect the Earth’s magnetic field in different directions, the solenoid was placed in three locations: in front, below, and to one side of the movement axis, and these locations were measured by the inertial sensors.

### 4.3. Experimental Validation

**Figure 5:**
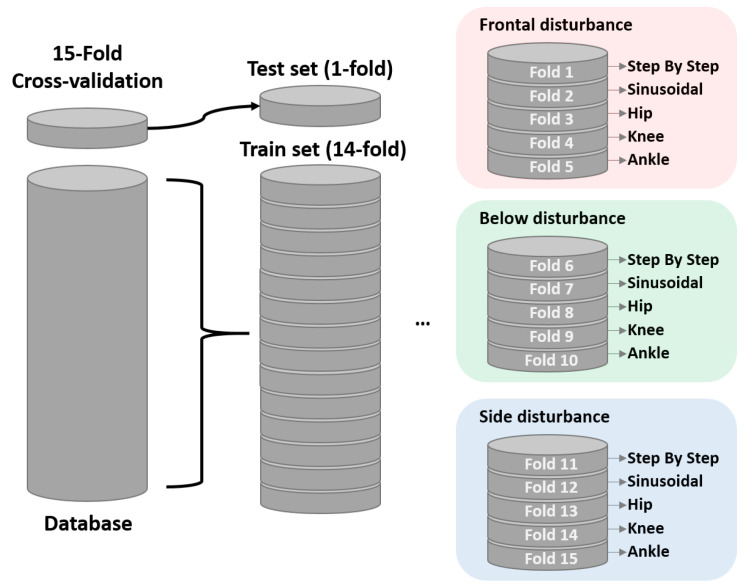
Distribution of the database into a nested 15-fold crossvalidation.

We adopted a crossvalidation scheme as proposed by [60], thus assuming different sets. We separated the sources of the data hiding one sensor, some types of signal, and the direction of the magnetic disturbance. Thus, we defined fifteen folds where the training set was completely independent of the test set. Figure 5 depicts in detail the distribution of each fold proposed in this experiment. This strategy is known as stratified crossvalidation.

Stratification seeks to ensure that each fold is representative of all types of data. Usually, this is done in a supervised way for classification and aims to ensure that each class is (approximately) equally represented across each test fold (and these classes are, of course, combined in a complementary way to form training folds). In our case, we ensured that the testing set did not have the same nature as the training data.

### 4.4. Performance Analysis

We present the results of our analysis, using the confusion matrix method, to calculate various metrics that evaluated the performance of our model. Since the purpose of this work is to detect magnetic disturbances, the positive class represents the state with magnetic disturbances, while the negative class represents the absence of magnetic disturbances (disturbance-free).

The true-positive rate (TPR), or sensitivity, is the ratio between the magnetic disturbed samples (MDSs) that have been correctly classified (true positives: tp) and the total number of MDSs (number of positives: Np). Notice that Np is equal to the sum of tp and false negatives (fn). The true-negative rate (TNR), or specificity, is the ratio between homogeneous magnetic field (HMF) files that have been correctly classified (true negatives: tn) and the total number of HMF files (number of negatives: Nn). Nn can be estimated by adding tn and false positives (fp). Finally, the system’s accuracy is the ratio between all the hits obtained by the system and the total number of samples. In summary we have the following:

(2) $$\begin{array}{cc} \; & Sensitivity=TPR=\frac{tp}{Np}=\frac{tp}{tp+fn},\\ \; & Specificity=TNR=\frac{tn}{Nn}=\frac{tn}{tn+fp},\\ \; & Accuracy=\frac{tp+tn}{Np+Nn}. \end{array}$$

In addition, we included the recall indicator, which measures the proportion of accurately predicted positive cases over the total number of positive cases in the data that the classifier was able to identify. The F1 score is a metric that combines recall and precision. Generally speaking, it is defined as the two’s harmonic mean. Simply put, the harmonic mean is an alternative method for calculating an average of numbers. It is generally accepted that this method works better for ratios (such as recall and precision) than the standard arithmetic mean.

Finally, the classification performance was measured at different threshold settings using the AUC–ROC curve. The AUC (area under the curve) is a metric or degree of separability, whereas the ROC (receiver operating characteristic) is a probability curve. It indicates the degree to which the model can discriminate between classes. The model performs better at predicting 0 classes as 0 and 1 classes as 1, thus indicating a higher AUC. By analogy, the model’s ability to discriminate between a magnetic field with disturbances and one that is disturbance-free is shown by the higher AUC.

## 5. Results

In this section, we present the results of our method alone and in comparison with the results obtained with a classical decision-making method based on a threshold. Finally, we show the effect of the temperature as a possible source of errors in the magnetic disturbance estimation.

### 5.1. Disturbance Detection Based on Deep Learning

In Table 1, we present the performance of the LSTM sequence-to-sequence architecture, considering the classifier behavior in each of the folds. We present the results in terms of accuracy (Acc), AUC, sensitivity (Sens), specificity (Spec), recall, and the F score. The results of the crossvalidation show that most of the folds achieved high performance, with accuracy and AUC values above 99% and 0.999, respectively. However, fold-1 and fold-2 were outliers, with their accuracy values dropping to the ranges of 85% and 92%, respectively, and their AUC values dropping to the ranges of 0.83 and 0.90, respectively. The only metrics that remained consistent across all folds were sensitivity and recall, which were close to 99%. The specificity and F score varied from 76% to 91% for fold-1 and fold-2, respectively.

**Figure 6:**
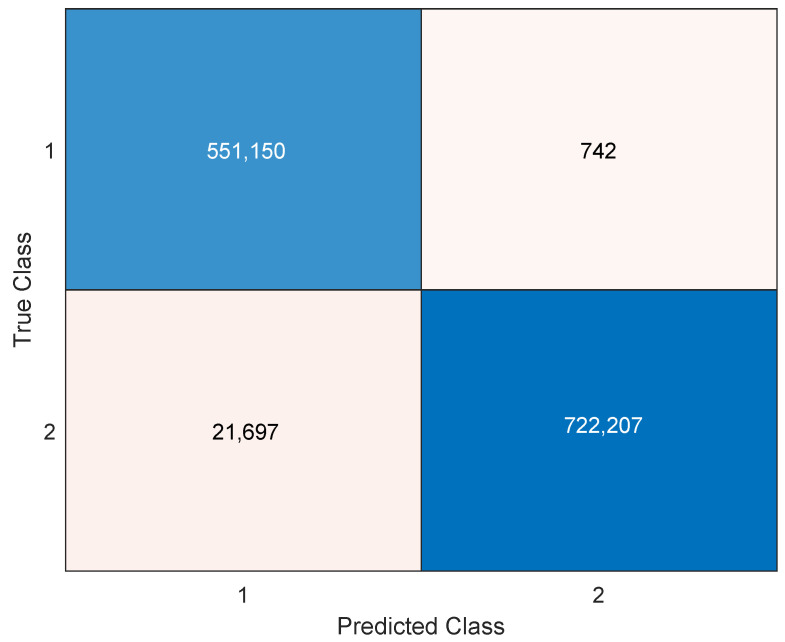
Confusion matrix of number of samples. Class 1 represents magnetic disturbed signals, and Class 2 represents no disturbed ones.

The confusion matrix from Figure 6 summarizes the results of the classification model for the two classes: Class 1, which indicates magnetic disturbance samples, and Class 2, which indicates magnetic disturbance samples. The columns show the predicted classes by the model, while the rows show the actual classes in the data. The diagonal elements (1,1) and (2,2) represent the correct predictions for each class, while the off-diagonal elements (1,2) and (2,1) represent the incorrect predictions for each class. For example, element (1,2) means that the model predicted Class 1 (magnetic disturbance) when the actual class was Class 2 (no magnetic disturbance).

### 5.2. Comparison with Threshold-Based Methods

Table 2 shows the results of threshold-based decision making in terms of accuracy, sensitivity, specificity, recall, and F score. For this experiment, we present the averages and their standard deviations, which were obtained from the test of all folds.

The results consider the threshold applied to the total magnetic field. We have evaluated two threshold cases. The first is Case I: the threshold was estimated from a time window where the magnetic field was not disturbed. The second is Case II: time window conditions were not controlled. There may be a combination of dynamic or static with disturbed or undisturbed. This means that the threshold may have had different levels of interference or stability, which can affect the performance of the decision.

### 5.3. Magnetic Disturbances Due to Changes in Temperature

**Figure 7:**
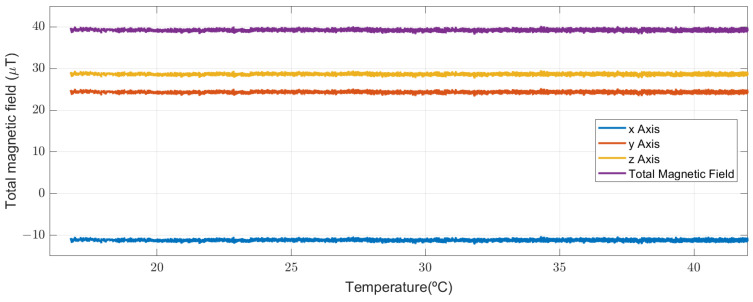
Performance of the magnetometer temperature compensation algorithm. We considered a temperature range between 16 and 45 degrees Celsius. The sensor remained in a temperature chamber without movement and with constant temperature change.

Temperature is a key factor, since it can generate a drift in the magnetometer measurement. However, this phenomenon has been widely addressed in the literature. The commercial device considered in this study already included a temperature compensation algorithm. Figure 7 shows the variation in the magnetic field, estimated with the magnetometer, as a function of temperature variation.

## 6. Discussion

We proposed a new methodology for the automatic detection of magnetic disturbances using MIMU sensors. We found an architecture capable of modeling our problem while avoiding the use of thresholds. To our best knowledge, there are no reported works that allowed for the detection of magnetic disturbances with high discriminant capacity based on purely magnetic information.

On average, the sequence-to-sequence classification process reached high performance values with an accuracy of 98.5% and with specificity and sensitivity values over 97%. In the same way, the recall and F score values showed high performance. When we checked fold by fold, we found that the sensitivity was very consistent, thus reflecting the increased capacity of the classifier to correctly detect a sample as a magnetic disturbance. Instead, the specificity showed greater variability, with the lowest value of 76% and a range of variability of 24 percentage points. This high variation represents the fluctuation in the correct detection of magnetic-free samples. Another performance indicator is the area under the receiver operating characteristic curve (ROC curve). In this respect, the folds has similar behavior with a deviation of less than 1%. The only value less than 90% was the fold-1, which was expected given its performance in terms of specificity.

We adopted a deep learning model that applied LSTM networks on a sequence-to-sequence classification problem. The main difference between our approach and the existing models is the use of two bidirectional LSTM layers, instead of one. We observed that this choice improved the classifier’s performance in terms of time and accuracy.

Since the accelerometer and gyroscope are inertial sensors, they only react to movements on these devices, not to changes in the magnetic field. Thus, we excluded them from the classifier inputs and used only the three-axis magnetometer information. This has the benefit of not relying on motions, and being applicable to both static and dynamic situations.

Another important result of this work is the generation of a controlled and reproducible dataset of magnetic disturbances, which allowed us to compare our algorithm with a threshold-based method proposed in the literature. This data set can be used, or reproduced, as a valuable reference for benchmarking.

The results, shown in Table 2, demonstrate the better performance and stability of our method in both static and dynamic conditions, thus highlighting the limitations of current methods. The performance of threshold-based methods is very sensitive to the time and place where the magnetic field measurement is performed in order to calculate the threshold. When this was calculated during undisturbed magnetic field conditions, as in Case I, the performance was higher than in dynamic conditions (Case II), where the threshold may have been defined in a disturbed magnetic field.

### Limitations and Future Work

Although our methodology demonstrated high accuracy in the automatic detection of magnetic disturbances, we must clarify that this type of architecture, based on bidirectional LSTM networks, does not allow for real-time applications. This is an intrinsic limitation of LSTM networks, which take advantage of the future of the signal to improve detection accuracy. An interesting future direction is to develop architectures that allow for real-time processing considering only past information in the time series.

This work has been motivated by the need to improve the performance of orientation estimation algorithms, mainly in applications involving human biomechanical analysis. For this reason, the angular motion generator considers human-like movements for reproducing those found in the most relevant lower limb human joints, such as the hip, knee, and ankle. However, this work does not rely on real human data, but considers only a 1-DOF circular motion. We did not consider high-frequency movements such as vibrations, because human movements, in general, do not have this characteristic.

This work is limited by measurements performed at constant temperature. However, as a mitigation to this limitation, we could verify that the sensor presents a stable behavior in the temperature range recommended by the manufacturer (16–45 degrees Celsius), with no deviations in the magnetic field estimation. This allowed us to rule out false positives in the detection caused by temperature changes.

Future work will consider the integration of this automatic magnetic disturbance detection in the orientation estimation processes of the motion capture system as a whole. The most important models of sensory fusion have specific modules for magnetic disturbance compensation. This integration could lead to significant improvements in disturbance compensation or rejection strategies.

## 7. Conclusions

This paper proposes a novel algorithmic approach for the estimation of magnetic disturbances in MIMU-based applications. Our main contribution resides in the development of a deep learning architecture that uses an LSTM sequence-to-sequence approach to model the detection of perturbed magnetic fields. Our model achieved an average accuracy of 98% under a wide variety of magnetic disturbances and motion dynamics. We used stratified folds to split the data, thereby ensuring that the training and test sets were independent and diverse. In this way, we reduced the network bias and obtained more trustworthy validation results.

In addition, we designed an experimental protocol for the generation of both magnetic field perturbations and MIMU motion patterns, which is in agreement with the benchmarking principles of repeatability, reproducibility, and rigor. The experiment simulated various realistic scenarios, thus allowing us to train a reliable discrimination model that can be deployed in real human motion analysis use cases.

These results confirm our ability to estimate orientation more precisely by considering the probability of sensing the magnetic disturbances and achieving high success rates. We believe that integrating our architecture in the state-of-the-art orientation estimation methods will improve the performance of current state-of-the-art motion analysis systems.

## Figures and Tables

**Table 1:** Results for automatic detection of magnetic disturbances. Each fold and average are shown.

	Direction	Signal	Acc (%)	AUC	Sens (%)	Spec (%)	Recall (%)	F Score (%)
Fold-1	Front	Step By Step	85.97	0.834	98.66	76.89	98.66	85.49
Fold-2		Sinusoidal	92.53	0.906	99.86	87.38	99.86	91.70
Fold-3		Hip	99.83	0.998	99.98	99.72	99.98	99.80
Fold-4		Knee	99.93	0.996	99.98	99.90	99.98	99.92
Fold-5		Ankle	99.88	0.997	99.98	99.81	99.98	99.86
Fold-6	Below	Step By Step	99.84	0.998	99.98	99.74	99.98	99.81
Fold-7		Sinusoidal	99.86	0.999	99.98	99.77	99.98	99.84
Fold-8		Hip	99.84	0.999	99.98	99.74	99.98	99.81
Fold-9		Knee	99.91	0.994	99.98	99.87	99.98	99.90
Fold-10		Ankle	99.84	0.993	99.98	99.73	99.98	99.81
Fold-11	Side	Step By Step	99.83	0.998	99.98	99.71	99.98	99.80
Fold-12		Sinusoidal	99.91	0.997	99.98	99.86	99.98	99.89
Fold-13		Hip	99.87	0.996	99.98	99.78	99.98	99.85
Fold-14		Knee	99.92	0.997	99.98	99.88	99.98	99.91
Fold-15		Ankle	99.88	0.996	99.98	99.80	99.98	99.86
Average			98.46	0.986	99.88	97.44	99.88	98.35

**Table 2:** Threshold decision-making methods.

	Acc (%)	Sens (%)	Spec (%)	Recall (%)	F-Score (%)
Case I	92.50 ± 10.37	98.99 ± 1.91	87.64 ± 16.80	98.99 ± 1.91	92.58 ± 10.50
Case II	86.99 ± 6.21	93.11 ± 12.55	81.25 ± 11.51	93.11 ± 12.55	86.28 ± 7.91

## Data Availability

The code and database may be shared upon reasonable request, only for research purposes, and not for industrial products.
